# Electrochemical Study of Ni20Cr Coatings Applied by HVOF Process in ZnCl_2_-KCl at High Temperatures

**DOI:** 10.1155/2014/503618

**Published:** 2014-08-25

**Authors:** J. Porcayo-Calderón, O. Sotelo-Mazón, M. Casales-Diaz, J. A. Ascencio-Gutierrez, V. M. Salinas-Bravo, L. Martinez-Gomez

**Affiliations:** ^1^Universidad Autonoma del Estado de Morelos, CIICAp, Avenida Universidad 1001, 62209 Cuernavaca, MOR, Mexico; ^2^Universidad Nacional Autonoma de Mexico, Instituto de Ciencias Fisicas, Avenida Universidad s/n, 62210 Cuernavaca, MOR, Mexico; ^3^Instituto de Investigaciones Electricas, Avenida Reforma 113, Colonia Palmira, 62490 Cuernavaca, MOR, Mexico; ^4^Corrosion y Proteccion (CyP), Buffon 46, 11590 Mexico City, DF, Mexico

## Abstract

Corrosion behavior of Ni20Cr coatings deposited by HVOF (high velocity oxygen-fuel) process was evaluated in ZnCl_2_-KCl (1 : 1 mole ratio) molten salts. Electrochemical techniques employed were potentiodynamic polarization curves, open circuit potential, and linear polarization resistance (LPR) measurements. Experimental conditions included static air and temperatures of 350, 400, and 450°C. 304-type SS was evaluated in the same conditions as the Ni20Cr coatings and it was used as a reference material to assess the coatings corrosion resistance. Coatings were evaluated as-deposited and with a grinded surface finished condition. Results showed that Ni20Cr coatings have a better corrosion performance than 304-type SS. Analysis showed that Ni content of the coatings improved its corrosion resistance, and the low corrosion resistance of 304 stainless steel was attributed to the low stability of Fe and Cr and their oxides in the corrosive media used.

## 1. Introduction

Corrosion is the major cause of downtime in garbage incineration installations plants and represents a large percentage of total maintenance costs of the plant. Corrosion in these areas is generally categorized as high-temperature corrosion [1]. In order to reduce the corrosion problems due to the operating conditions of the boilers, there are different primary methods, such as improving the combustion processes and improving the control processes including the control of gas temperature, and changes to the boiler design as gas recirculation to alter the flow dynamics and promote mixing gas. There are also secondary methods to extend the lifetime of the boiler tubes, such as the use of coatings to protect the pipes from corrosive deposits and gas flows, the use of more corrosion resistant alloys, the combined use of protective coatings and more resistant alloys, or the use of either corrosion inhibitors or refractory coatings in the lower parts of the combustion chamber [2]. However, even though different methods have been implemented in order to reduce corrosion problems, this phenomenon still exists and it is a problem to be analyzed continually.

When a molten salt is present, the salt wets the oxide surface and is able to penetrate through the pores and cracks by capillary action. Transport by diffusion through the molten salt is much faster than solid state diffusion. Therefore, the superficial layer will grow much faster and the important chemical reactions will be those involving the phases present in the molten salt. The molten salts provide a means for the transport of both the oxidant to the metal and dissolved metal ions to the outside. The molten salts are able to permeate through the porosity of the oxide layer and a wide range of local activities of oxygen from oxidizing to reducing conditions can be found. The reactions that form metallic chlorides near the metal-oxide interface will consume the alloy components responsible for repairing the protective oxide scale. Metallic chlorides, which can be formed above or below the oxide-metal interface, can migrate to the melt-gas interface as dissolved species. Because of their porous nature, they can precipitate as nonprotective oxides providing a route for the continuous penetration of salt and gaseous species [3]. Better understanding of mechanisms of material degradation by salt compounds is an important issue to reduce tube consumption rate in waste incinerators. Coatings are an alternative to manage the corrosion of tube materials. This approach is a feasible alternative to increase the service life of materials [2]. This investigation shows the results of the evaluation of Ni20Cr coatings deposited by HVOF process in a ZnCl_2_-KCl eutectic mixture.

## 2. Experimental Procedure

### 2.1. Materials

A Ni20Cr (%wt.) powder alloy was used as coating material. Coatings were applied on 0.25 inches diameter AISI 304-type stainless steel rods (304 SS) by HVOF process using a Sulzer Metco model Diamond-Jet system. In all cases, the same working conditions such as distance of spraying, pressure, and flow of gases were maintained for all coatings. Before coating, all rods were cleaned with acetone and their surface was shot blasted with alumina particles according to NACE number 1/SSPC-SP 5 recommended practice [4]. After shot blasting, specimens were cleaned again with acetone and were ready for coating. For corrosion tests the coated specimens were used with two surface conditions: as-deposited and grinded with 600-grade emery paper. In order to avoid the presence of galvanic corrosion by the diffusion of molten salts to the substrate, coating thickness deposited was at least 500 microns.

### 2.2. Electrochemical Measurements

The electrochemical behavior was studied using potentiodynamic polarization curves (*E*_corr_ versus *I*_corr_), open circuit potential (*E*_corr_), and linear polarization resistance (LPR) measurements. Polarization curves were measured by scanning the potential from −400 to 1500 mV applying a scanning rate of 1 mV/s, and Tafel slopes (*β*_*a*_, *β*_*c*_) were obtained from the active regions of the corresponding anodic and cathodic curves. To determine LPR, a potential polarization of ±10 mV was applied and current intensity associated with that polarization was measured hourly up to a total of 48 hours. Electrochemical tests were carried out using an ACM Instruments Auto-DC potentiostat, controlled by a personal computer.

**Figure 1:**
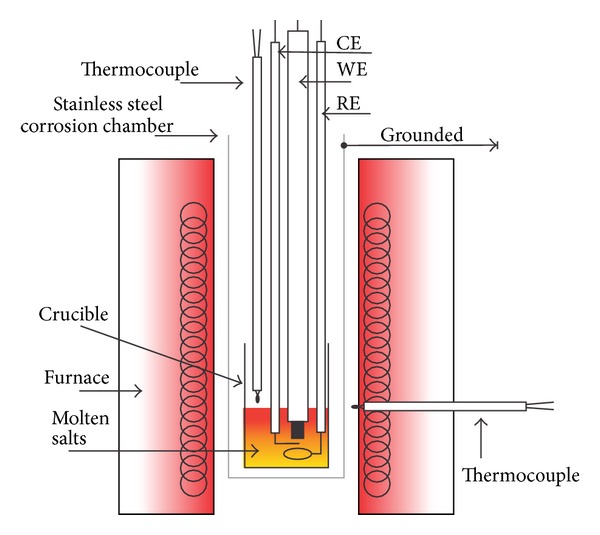
Experimental setup of the electrochemical cell.

The electrochemical cell was based on a three-electrode system which includes one working electrode (WE), one reference electrode (RE), and one counter electrode (CE). The 304 SS and coated specimens were used as the working electrodes. Two platinum wires (0.5 mm diameter) inside a mullite tube were used as reference electrode and counter electrode. For electrical connection, working electrodes were spot-welded to a Ni20Cr wire. Ceramic tubes were used for isolating the electrical wire from the molten salt and the gap between the ceramic tube and electrical connection wire was filled with refractory cement. A 20 mL alumina crucible was used for containing the corrosive mixture and placed inside an electrical furnace. A closed end and grounded stainless steel tube was used as the corrosion chamber to eliminate the effect of electric and magnetic fields induced by the furnace on electrochemical measurements. The corrosion mixture (KCl-ZnCl_2_) was prepared with analytical grade reagents. Dried chloride salts were first weighted in desired 1 : 1 mole ratio (240°C melting point) and then subjected to a mechanical milling in an agate mortar to obtain well-mixed reagents. Test temperatures were 350, 400, and 450°C. When the test temperature was stabilized, the three electrochemical cell electrodes were introduced inside the molten salt. In all experiments the atmosphere above the melt was static air. Experimental setup used in the present study is shown in Figure 1.

After testing, the electrode used as working electrode was mounted in bakelite in cross-section and polished to analyze the subsurface corrosive attack using a scanning electron microscopy (SEM) aided with X-ray energy dispersive spectroscopy (EDS) to carry out microchemical analysis.

## 3. Results and Discussion

### 3.1. Coating Features

**Figure 2:**
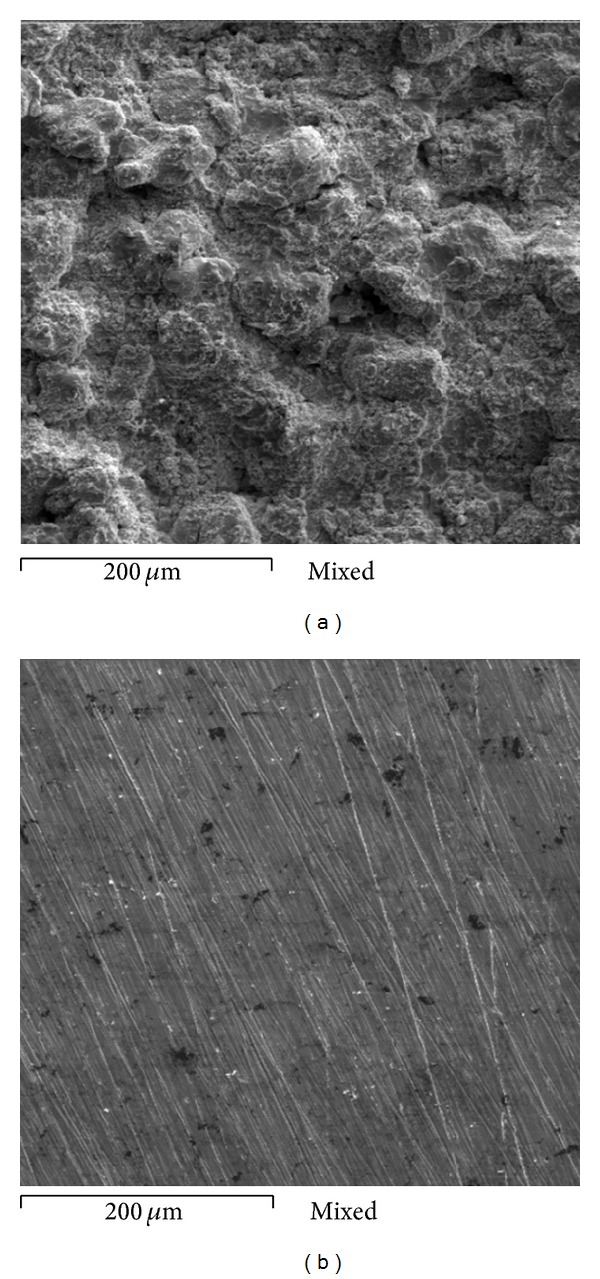
SEM micrographs showing characteristics of the coatings; (a) as-deposited surface and (b) grinded surface.

**Figure 3:**
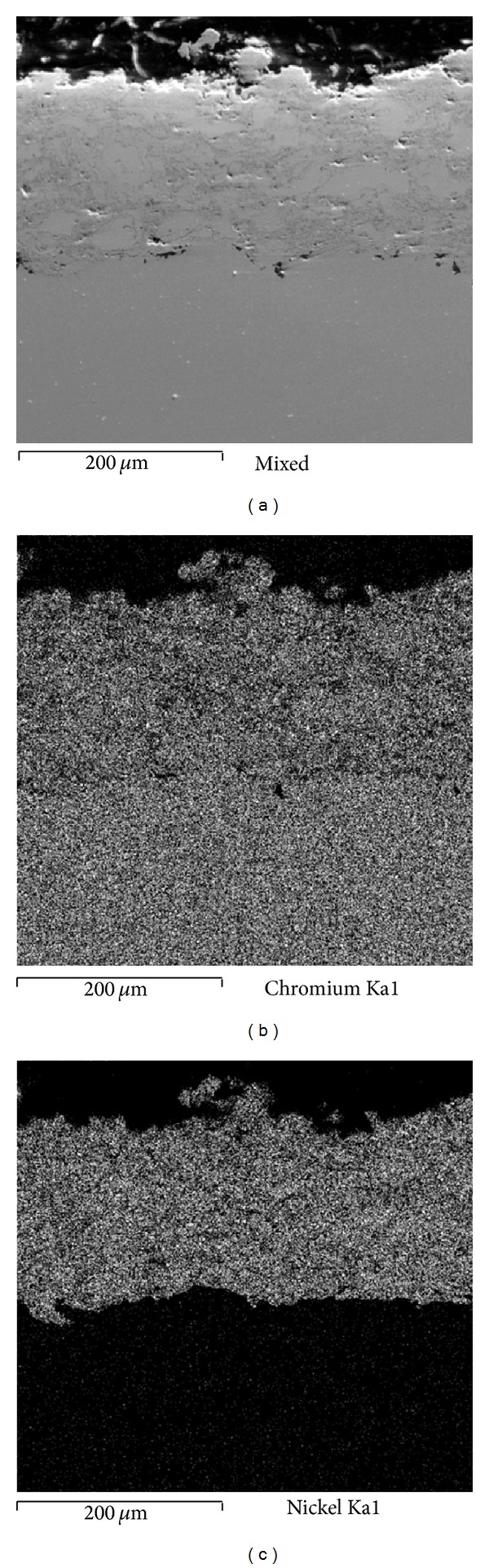
Cross-section aspect of the coatings and elements mapping.

Figure 2 shows different aspects of the Ni20Cr coatings tested.  Figure 2(a) corresponds to the as-deposited superficial condition. An irregular surface without apparent porosity and with dense aspect is observed contrary to that observed in Ni20Cr coatings applied by powder thermal spray process [5]. It is known that in its as-deposited condition the coated surface is oxidized because of the spraying process. Furthermore, due the surface roughness is difficult to measure the real area of the working electrode, and thus the values obtained from electrochemical measurements can be lower than those reported. Therefore, in order to homogenize the surface and remove the external oxidized layer the coatings were also evaluated in the grinded surface condition, Figure 2(b). In this case a homogeneous surface without porosity and with denser aspect is observed. Some dark phases are observed which correspond to chromium oxides formed during the projection process [5].  Figure 3 shows the cross-section of Ni20Cr coatings and elements mapping and the presence of low porosity, dense aspect, and homogenous elements distribution is observed. All these characteristics are typical of the coatings deposited by the HVOF process [6].

### 3.2. Potentiodynamic Polarization Tests

**Figure 4:**
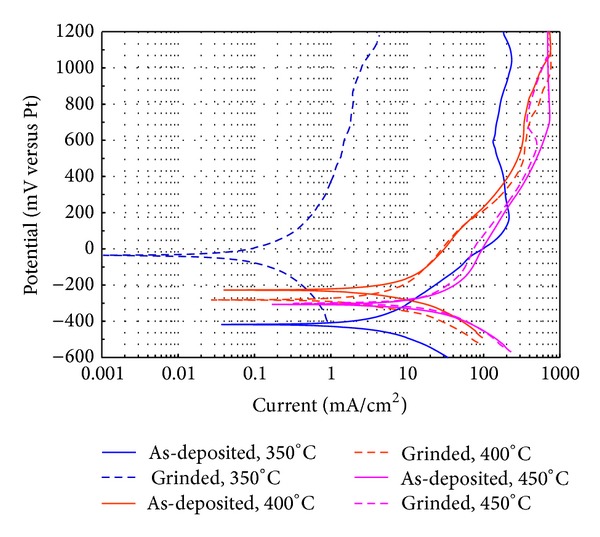
Polarization plots of Ni20Cr coatings in KCl-ZnCl_2_ at the different temperatures.

Figure 4 shows the potentiodynamic polarization curves of Ni20Cr coatings evaluated in ZnCl_2_-KCl at different test temperatures. Ni20Cr coatings in the as-deposited condition at 350°C show a more cathodic *E*_corr_ (−418 mV) at 400°C and 450°C and the *E*_corr_ is noblest (−227 mV and −306 mV, resp.). Branches of the anodic polarization curves only show the presence of a passive zone at 350°C, and at 400°C and 450°C a continuous dissolution process is observed. *I*_corr_ values observed increase exponentially with respect to the test temperature. Absence of a passive zone and an exponential increase of the *I*_corr_ may be because the thickness of the oxidized top layer is smaller than those of the Ni20Cr coatings applied by powder thermal process [5]. Regarding Ni20Cr coatings in the as-grinded condition, the corrosion potential behavior (*E*_corr_) becomes more cathodic with the test temperature, being −35, −282, and −302 mV at 350°C, 400°C, and 450°C, respectively. The anodic branches do not show a tendency to establish a passive zone. Though being in as-grinded condition, both roughness and surface oxidized layer were eliminated; the polarization curves at 400°C and 450°C either as-deposited and as-grinded conditions are practically identical. *I*_corr_ values also increase exponentially regarding the test temperature. *I*_corr_ values are smaller than those observed in as-deposited condition. It can be due to the fact that the calculated reaction area is closer to the actual reaction area.  Table 1 shows the electrochemical parameters determined from polarization curves of Ni20Cr caotings and 304 SS. Comparing the electrochemical parameters of Ni20Cr coatings regarding 304 SS the following features arise. As expected, the *I*_corr_ values of each material increase with a temperature increase. *I*_corr_ values of 304 SS for all temperatures are less than *I*_corr_ values of Ni20Cr coatings in the as-deposited and grinded conditions. Comparing both coating conditions, *I*_corr_ values of the grinded conditions are less than *I*_corr_ values in the as-deposited condition.

According to Table 1 at 350°C both 304 SS and as-grinded coating had the greater corrosion resistance, but at 400°C and 450°C the corrosion resistance of 304 SS was better. However, it should be noted that this behavior corresponds to the beginning of the corrosion process. This trend may change in long-term exposures because either the corrosion process can induce changes in the chemistry of the molten salts, making them more aggressive, or the material may not be able to regenerate protective oxide layers.

### 3.3. Free Corrosion Potential Curves

**Figure 5:**
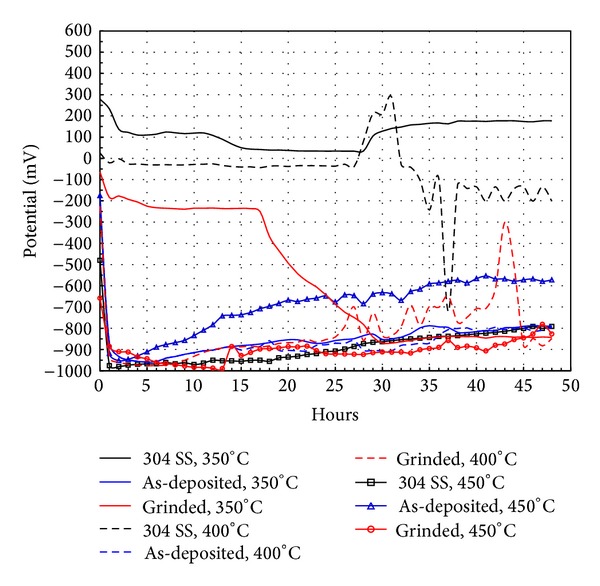
Evolution of *E*_corr_ values as function of testing time for the different materials.

Electrochemical methods are extremely useful in studying corrosion processes. The variation of the open circuit potential of a surface in an electrolyte provides information about the evolution of the surface, that is, active or passive behavior. *E*_corr_ as a function of time for the different materials tested in ZnCl_2_-KCl molten salts is shown in Figure 5. One simple way to study the film formation and passivation of materials immersed in molten salts is by monitoring *E*_corr_ as a function of time. A rise of potential in the positive direction indicates formation of a passive film and a steady potential indicates that the film remains intact and protective. A drop of potential in the negative direction indicates breaks in the film, dissolution of the film, or no film formation. The test temperature significantly affects the *E*_corr_ alloys behavior. Usually an increase in temperature increases the aggressiveness of the molten salts and corrosion resistance will depend on the protective capacity of the oxides formed on the alloys. It is observed that, increasing temperature, *E*_corr_ values of 304 SS become more active. At a temperature of 350°C, *E*_corr_ decreases up to 28 hours and then tends to increase until the end of the test. This behavior indicates that 304 SS initially experienced a slight corrosion process and subsequently was able to form a protective oxide (Cr_2_O_3_) that protected it from the action of molten salts. At 400°C *E*_corr_ remains almost constant (−25 mV) during 27 hours and then it shows abrupt changes until the end of the test. This may be associated with the dissolution of protective oxide layers due to the action of molten salts. At 450°C in the first hour of testing, there are a sharp drop of *E*_corr_ from −500 mV to −950 mV and then a slow increase to −800 mV until the end of the test. This indicates a strong attack of the material and a subsequent attempt to form a protective oxide. Another explanation could be the accumulation of corrosion products which prevented the free molten salts access to the corroded surface. Trend *E*_corr_ values for the Ni20Cr coatings in either as-deposited or grinded condition were similar at all temperatures tested. *E*_corr_ values show a sharp drop in the first 3–5 hours and then a further increase until the end of the test. The initial behavior can be associated with the dissolution process of the surface oxides and an attempt of self-healing. However, at 350°C Ni20Cr coating as-grinded showed a slow drop in *E*_corr_ values until 17 hours, a fast drop until 30 hours, and then a similar behavior to the other coatings until the end of the test.

### 3.4. Linear Polarization Curves

**Figure 6:**
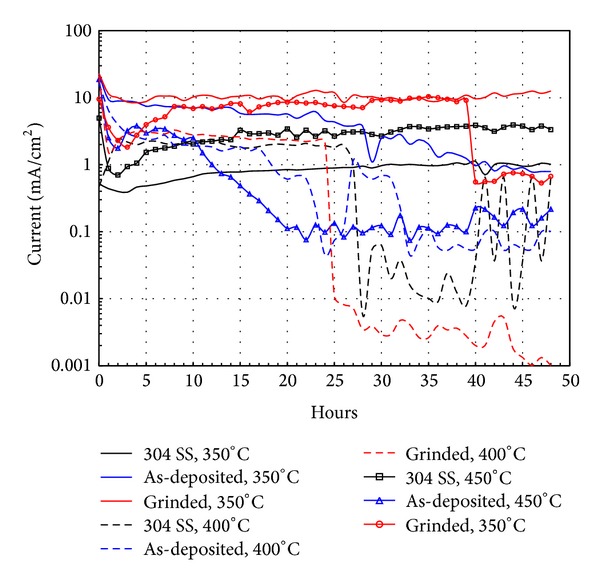
Current density (*I*_corr_) plots of different materials at different temperatures.

**Figure 7:**
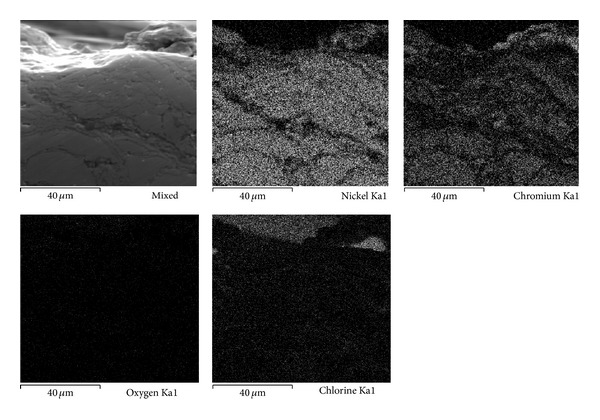
Cross-sectional aspect of the Ni20Cr as-deposited coating and elements distribution after corrosion test at 350°C.

Figure 6 shows the evolution of the *I*_corr_ obtained by linear polarization measurements over time for different materials tested. It is known that once polarization resistance is determined, calculation of *I*_corr_ requires knowledge of the Tafel constants, and these constants can be determined from experimental polarization curves. Also, in the absence of these values, an approximation is sometimes used, and the expected error in the calculated value of *I*_corr_ should be less than a factor of two [7]. However, when the results show polarization resistance values within the same order of magnitude, it is necessary to use more precise values of the Tafel slopes in order to perform a reliable analysis of the results [8]. Therefore, the values shown in Figure 7 were obtained from the polarization resistance measurements using Stern-Geary expression [7]. Consider

(1) $$\begin{array}{c} I_{\text{corr}}=\frac{\beta_{a}\beta_{c}}{2.3R_{p}\left(\beta_{a}+\beta_{c}\right)}, \end{array}$$

where the *β*_*a*_ and *β*_*c*_ values were those reported in Table 1.

At 350°C, 304 SS showed an almost constant *I*_corr_ between 0.5 and 1.0 mA/cm^2^. At 400°C, an increase in *I*_corr_ from 0.5 to 2.2 mA/cm^2^ in the first two hours of immersion, remaining constant up to 26 hours, and then decrease to very low values until the end of the test were observed. At 450°C the 304 SS shows that *I*_corr_ decreased during the first hour of immersion and then tended to increase steadily to the end of the test to *I*_corr_ values around 4 mA/cm^2^. This behavior shows that initially the material was protected by a protective oxide layer, but this one was not stable because *I*_corr_ was always increasing. Concerning Ni20Cr as-deposited coatings, Figure 6 shows that the *I*_corr_ variation showed the same behavior at all test temperatures; that is, *I*_corr_ tended to decrease throughout the test period. These decreases in the *I*_corr_ values were greater with increasing temperature. This behavior can be explained because the growth rate of the protective oxide is enhanced by increasing the temperature. Analyzing the Ni20Cr coating in the as-grinded surface condition, at 350°C, it showed a steady behavior in its *I*_corr_ values around 10–12 mA/cm^2^, but these were the largest of all the materials evaluated. This behavior is consistent with a coupled process of growth and dissolution of the protective oxide. At 400°C, during the first hour of testing, there is a sharp decrease in *I*_corr_ values from 21 to 1.5 mA/cm^2^, later showing a steady increase until 2 mA/cm^2^, and then a sharp drop was observed until values around 0.01 mA/cm^2^. This indicates that the coating had the ability to self-heal because of the growth on its surface of a protective oxide layer. At 450°C during the first 2 hours of immersion, a decrease in the *I*_corr_ values from 10 to 2 mA/cm^2^, further a steady increase to 10 mA/cm^2^, and then a sharp drop to values around 1 mA/cm^2^ were observed. In all cases the highest *I*_corr_ values observed during the first hours of immersion are due to the dissolution of protective oxides by the corrosive action of molten salts [9]. Initial trend in the *I*_corr_ values for the Ni20Cr coatings in either as-deposited or grinded condition showed a sharp drop in the first 2 hours and this behavior can be associated with the fast growth of protective oxide layer that prevented the penetration and attack of the molten salts.

### 3.5. SEM Analysis

Although the electrochemical methods are extremely useful in studying corrosion processes, they alone do not provide enough information to elucidate the mechanism of the system under study. Therefore the use of complementary techniques, that is, scanning electron microscopy (SEM) and auger electron spectroscopy (AES), among others, has been suggested in order to clarify both the morphology of the attack and the chemical composition and distribution of the elements present. Combination of these methods provides the information to understand the reactions occurring on the surface [10]. Therefore in this study the cross-section of the working electrodes was studied using scanning electron microscopy (SEM) to clarify both the morphology and the elements distribution. Scanning electron microscopy enables visualization of the morphology of the attack of the surface and identification of parts that show compositional differences. With high-resolution Auger spectroscopy it is possible to detect the elements present on a surface. Moreover, it is possible to track differences in the chemical state of the elements found in different places on the surface. A combination of this technique and an ion gun also provides information about the in-depth evolution of the surface composition.

**Figure 8:**
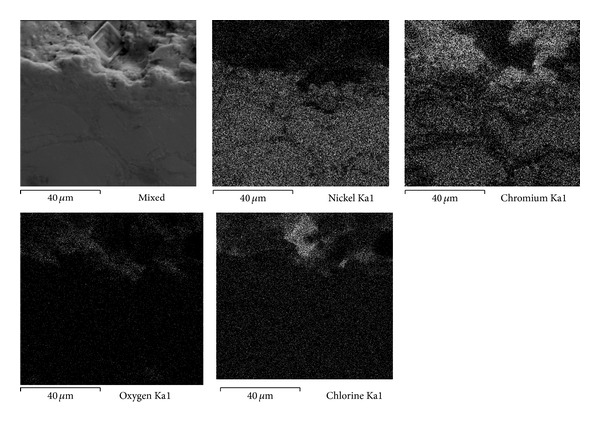
Cross-sectional aspect of the Ni20Cr as-deposited coating and elements distribution after corrosion test at 400°C.

**Figure 9:**
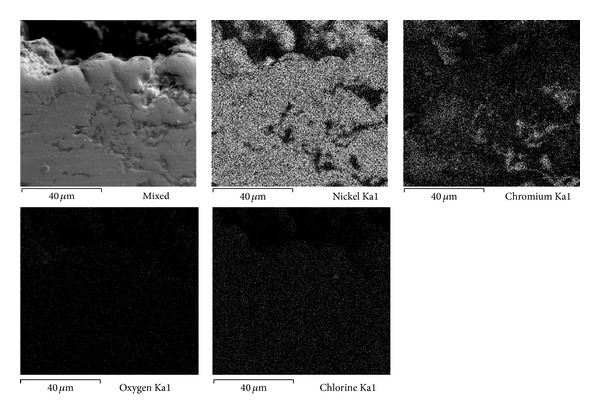
Cross-sectional aspect of the Ni20Cr as-deposited coating and elements distribution after corrosion test at 450°C.

**Figure 10:**
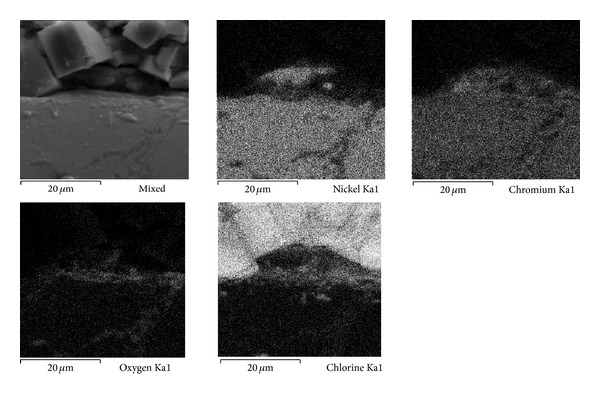
Cross-sectional aspect of the Ni20Cr as-grinded coating and elements distribution after corrosion test at 350°C.

**Figure 11:**
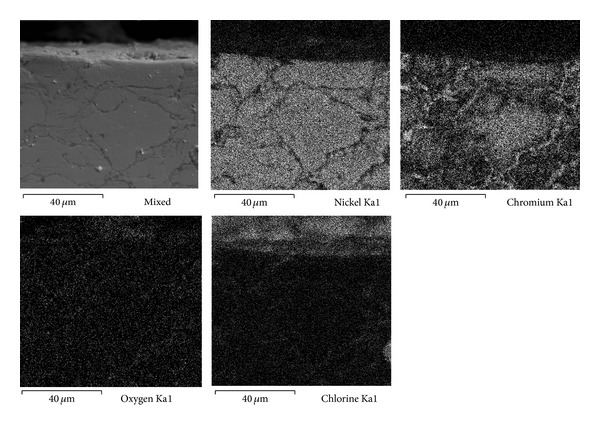
Cross-sectional aspect of the Ni20Cr as-grinded coating and elements distribution after corrosion test at 400°C.

**Figure 12:**
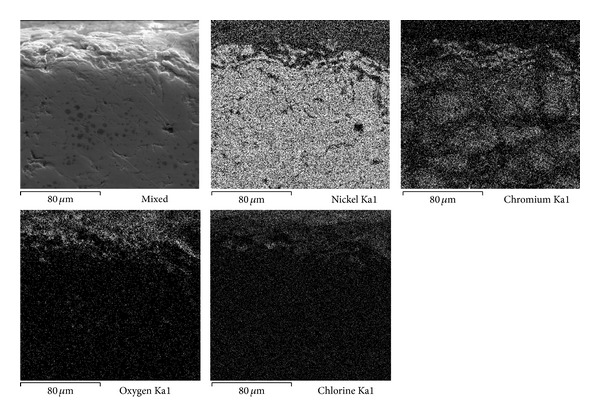
Cross-sectional aspect of the Ni20Cr as-grinded coating and elements distribution after corrosion test at 450°C.

Figures 7, 8, and 9 show the characteristics of Ni20Cr coatings in the as-deposited conditions showing the elements distribution after the corrosion test. It is observed that at 350°C the coating remained virtually unchanged on its surface, and it only shows the presence of corrosive agent on its surface. EDS analysis showed a Cr content of 18.6% near the coating surface. In the same way at 400°C the coating surface remained unchanged and molten salts slightly penetrated through the coating surface porosity and no significant degradation is observed. EDS analysis showed a Cr content of 20.3% near the coating surface. At 450°C a surface deterioration of the coating where the molten salts were able to penetrate lightly into the coating structure is observed. EDS analysis showed a Cr content of 7.6% near the coating surface. The biggest attack was observed at this temperature, where the Cr reacted preferably with ions chlorine. According to the mapping elements this allowed a Ni enrichment in the external layers of the coating. Analyzing the Ni20Cr coating in the as-grinded surface condition, at 350°C and 400°C (Figures 10 and 11) it is observed that the coating surface remained unchanged, and only the presence of corrosive agent on its surface was observed. This indicates that coating had the ability to self-heal because of the growth on its surface of a protective oxide layer. At 450°C (Figure 12) a slight surface degradation of the coating was observed and molten salts penetrated through its porosity which caused the observed increase in *I*_corr_ values.

In both cases it was noted that coatings degradation starts to be significant at 450°C. In all cases the protective oxide is the chromium oxide and the attack of the materials began according to the following reaction:

(2) $$\begin{array}{c} {\text{Cr}}_{2}{\text{O}}_{3}+3{\text{Cl}}_{2}=2\text{Cr}{\text{Cl}}_{3}+\frac{3}{2}{\text{O}}_{2} \end{array}$$

This reaction suggests that chromium oxide is converted into metallic chloride in environments with low oxygen partial pressure; that is, this can only occur at the interface of metal-molten salt. The metallic chlorides have high vapor pressures and can spread easily toward regions with higher oxygen partial pressure where the reverse reaction is favored; that is, metallic chlorides are oxidized to form no protective metallic oxides. Similar reactions occur for the case of both Fe and Ni oxides to form FeCl_2_ and NiCl_2_. However, it is known that NiCl_2_ has a higher thermodynamic stability and a lower vapor pressure compared with iron and chromium chlorides at the same temperature, and the solubility measurements of oxide scales in Cl-rich molten salts showed that NiO is less soluble than the Fe and Cr oxides [11].

**Figure 13:**
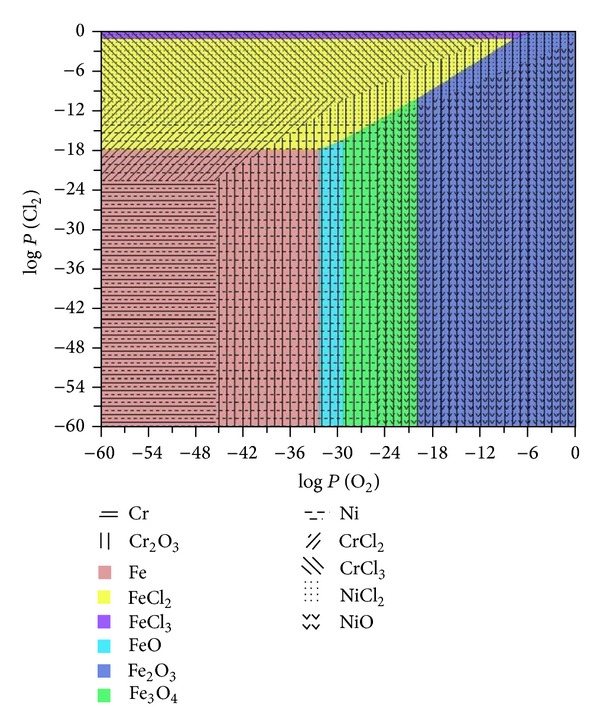
Thermodynamic stability diagrams for Fe-Cr-Ni-Cl-O at 450°C.

Results imply clearly that a high Ni content is very effective in improving the corrosion resistance while Cr plays a detrimental role under the same conditions. Corrosion protection of any material in molten salts depends on the chemical stability of both the metallic elements and their compounds such as oxides and chlorides. Regarding this, Figure 13 shows the phase stability diagram for the Fe-Cr-Ni-Cl-O system at 450°C. The procedure to generate the diagram is described elsewhere [12]. It is observed that from the thermodynamic viewpoint Ni is the most stable material. Therefore, Ni will remain immune in O_2_ and Cl_2_ environments where the Fe and Cr would be corroding continuously. In contrast, analyzing the oxides it is observed that Cr_2_O_3_ is the most protective oxide because its stability boundary is located at partial pressures of O_2_ lower than that of Ni and Fe oxides. Therefore, according these observations, it can be assumed that the NiCr-based alloys or coatings will have a better performance than Fe-base alloys. This combined effect has been established in other studies [9, 13–16]. Therefore at temperatures until 450°C, Ni-rich coatings will show better performance compared with those alloys richer in Fe or Cr. This analysis is consistent with reported studies where they indicate that Ni or its alloys show better performance in chlorides-rich environments compared with Fe and Cr or their alloys [15, 17, 18].

## 4. Conclusions

Corrosion protection of any material in molten salts depends on the chemical stability of both the metallic elements and their compounds such as oxides and chlorides. The results showed that Ni20Cr coatings have higher corrosion resistance than 304 stainless steel in molten ZnCl_2_-KCl at all temperatures evaluated. The superficial condition and the spray process are very important in the corrosion resistance of the coatings. HVOF process develops coatings with highest density, lowest surface porosity, and high bond strength. In this study the results imply clearly that a high Ni content is very effective in improving the corrosion resistance while Cr and Fe play a detrimental role. Therefore Ni-rich coatings will show better performance compared with those alloys richer in Fe or Cr.

## Figures and Tables

**Table 1:** Electrochemical parameters of potentiodynamic polarization tests.

Materials		*E*_corr_ (mV)	*β*_*a*_	*β*_*c*_	*I*_corr_ (mA/cm^2^)
304 SS	350°C	−202	137	174	0.39
	400°C	−77	300	277	2.03
	450°C	−141	188	118	1.89

As-deposited coating	350°C	−418	296	182	4.23
	400°C	−227	444	219	9.16
	450°C	−306	698	300	36.19

Grinded coating	350°C	−35	600	565	0.24
	400°C	−282	403	177	6.13
	450°C	−302	638	292	28.45
